# A focus on coordination chemistry at chlorine

**DOI:** 10.1039/d3sc90047a

**Published:** 2023-03-28

**Authors:** Jason L. Dutton

**Affiliations:** a Department of Chemistry, La Trobe Institute for Molecular Science, La Trobe University Melbourne Victoria Australia j.dutton@latrobe.edu.au

## Abstract

The first crystallographic characterization of chloronium cations stabilized by pyridine ligands (P. Pröhm, W. Berg, S. M. Rupf, C. Müller and S. Riedel, *Chem. Sci.*, 2023, https://doi.org/10.1039/D2SC06757A) is discussed in the context of coordination chemistry at chlorine.

## Abstract 2

A highlight of the recent report on the remarkable outcomes of the seemingly simple reaction of pyridines with chlorine gas.

## Graphical abstract

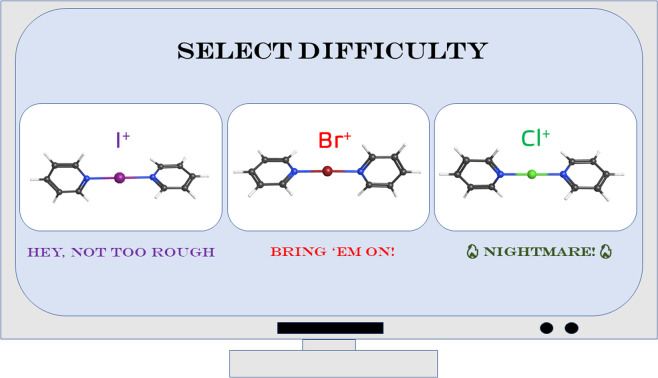

Coordination chemistry has most frequently been associated with classic transition metal–ligand complexes, where ligands donate two electrons to metals to form sigma bonds, often designated as coordinate or dative bonds. There is also rich coordination chemistry in the main group, with a classic example being the many adducts to boron or the heavier, more metallic, p-block elements.

Generally, more electronegative atoms are more electron rich and less likely to act as Lewis acids. The halogens (F, Cl, Br, I) are an electronegative group of elements and their chemistry is dominated by gaining electrons and existing in the −1 oxidation state. As such there is relatively less coordination chemistry for the halogens as compared to the other groups, nonetheless they do act as Lewis acids in compounds where they are found in higher oxidation states. Charge transfer complexes of the type Nu–X–X are well-studied.^1^ They are most stable and common for iodine and several compounds have been crystallographically characterized, including for pyridine^2^ and phosphine ligands,^3^ for example. They are less common for bromine but can be observed^4^ and in more rare cases isolated.^5,6^ For chlorine there are few examples where the charge transfer complex can be observed,^7^ although they have been well studied computationally.^8^

Unsurprisingly, iodine as the least electronegative member of the group (radioactive astatine aside) has the best known and most stable coordination compounds where the halogen is found in the higher +1 oxidation state. The easy synthesis of [bis(pyridine)iodine][nitrate] ([Pyr-I-Pyr][NO_3_]), from I_2_, pyridine and AgNO_3_ has even been implemented as undergraduate experiments at some institutions. The [BF]_4_^−^ salt, known as Barluenga’s reagent, is commercially available and widely used.^9^ The bromine analogue has been known for a very long time and has been crystallographically characterized,^10–12^ but rarely used, with a SciFinder search returning only a few dozen papers over 60 years compared to a few hundred for iodine. The chlorine analogue was first detected in solution at −80 °C by Erdélyi and co-workers in 2014 and they found that, as predicted by theoretical studies, the analogue forms a symmetric [Pyr–Cl–Pyr]^+^ species.^13^ [Pyr–F]^+^ is a commercially available electrophilic fluorinating reagent and Erdélyi found that addition of a second pyridine to this compound resulted in an asymmetric environment at low temperature. Additionally our group found that attempts to observe any bis-pyridine adduct at room temperature, or perform ligand exchange, resulted in decomposition to complex mixtures.^14^

Which brings us to the remarkable recent results from Riedel and co-workers who were able to crystallographically characterize both mono and bis-pyridine adducts of chloronium cations [Cl]^+^ as well as a pyridine–Cl–Cl charge transfer complex for the first time.^15^

For the syntheses, the problem of obtaining stoichiometric amounts of Cl_2_ gas was solved by first condensing and weighing Cl_2_ into pressure tubes. This was then condensed onto the appropriate amount of pyridine or lutidine in propionitrile at −196 °C. Solutions were warmed to −40 °C to allow for reactions and cooled back to −80 °C to obtain crystals for X-ray analysis. The care required to achieve this is remarkable!

For the reactions of pyridine and lutidine with Cl_2,_ differing results were observed. For pyridine a Pyr–Cl–Cl complex was crystallized, while for lutidine, a [Lut–Cl–Lut][Cl_3_] salt was obtained. This is a good illustration of the divergent results that can be obtained with subtle changes in systems containing weak bonds. The reactions begin with pyridine interacting with the σ* orbital of chlorine, forming the complex observed for pyridine. This also induces weakening and polarization of the Cl–Cl bond. For the slightly stronger Lewis base lutidine, the polarization likely becomes strong enough to allow for another Cl_2_ to abstract a chloride, and onwards reactivity to the cationic complex.

Polyhalogen anions are not ideal counterions as they bring substantial reactivity into systems. For the iodine and bromine analogues this issue was solved using Ag^+^ cations to further polarize the Pyr–X–X complex, precipitating out AgX and delivering the chosen counterion paired with the silver cation. Using this strategy in the Cl^+^ system with AgBF_4_ generated [Pyr–Cl–Pyr][BF_4_] but also the salt [Ag(Pyr)_4_][BF_4_]_2_. Silver has been oxidized to Ag(ii), not a favoured oxidation state, which is indicative of these systems being highly oxidizing. Use of NaBF_4_, where Na^+^ cannot be further oxidized circumvented this issue, and [Pyr–Cl–Pyr][BF_4_] is the sole product observed. In all cases the N–Cl–N bonds show a shorter bond and a longer bond, but in solution the compounds appear symmetrical as previously determined,^13^ and theoretical calculations also indicate identical N–Cl bonds in the geometric minima. The asymmetry likely arises from solid state packing effects and this has a substantial influence due to the weak nature of the bonds in these complexes. All of the compounds described decompose if exposed to a temperature of −10 °C.

The team attempted to generate a monopyridine chloronium complex *via* abstraction of Cl^−^ from Pyr–Cl–Cl with a variety of halide abstraction agents, but this resulted in complex mixtures. Aryl groups are often susceptible to decomposition in the presence of strong halogen electrophiles, and this is likely the mode of decomposition here. To achieve the target complex, pentafluoropyridine was employed, where the perfluorination protects the ring from electrophilic aromatic substitution reactions, alongside [Cl_2_F][AsF_6_] as the Cl^+^ source. This exotic salt, previously known but not structurally characterized until this report, was generated from ClF and AsF_5_ in anhydrous HF that had been dried (repeatedly) with elemental F_2_. The skills and techniques required to do this chemistry are becoming a dying art and this synthesis is likely not achievable in many laboratories, but it does nicely illustrate the lengths that researchers need to go to in the name of inventing new chemistry! The N–Cl bond in the perflourinated [Pyr–Cl]^+^ cation is shorter than the corresponding bond for normal pyridine (1.69 Å *vs.* 1.76 Å). In the report this was indicated as surprising due to the weaker Lewis basicity of fluoropyridine, but it is likely due to the *trans* effect of the polarized chlorine being greater than the *trans* effect exerted by weakly coordinating [AsF_6_]^−^. Like solid-state effects, *trans* effects have an outwardly substantial influence in systems containing weak bonds.

In summary, this discovery by Riedel and co-workers is an outstanding example of the exquisite care and skill sometimes needed to bring something appearing so simple (pyridine + Cl_2_!) to the light of day. It will be exciting to see how this chemistry can be developed further, potentially for example as a source of electrophilic chlorine, or if derivatives/conditions can be found that allow for the “bottling” of such chlorine cations, for convenient storage and later use.

## Author contribution

Article written by J. Dutton.

## Conflicts of interest

There are no conflicts to declare.

## Supplementary Material
